# Power and sample size estimation for epigenome-wide association scans to detect differential DNA methylation

**DOI:** 10.1093/ije/dyv041

**Published:** 2015-05-12

**Authors:** Pei-Chien Tsai, Jordana T Bell

**Affiliations:** Department of Twin Research and Genetic Epidemiology, King’s College London, London, UK

**Keywords:** EWAS, power, DNA methylation, epigenome-wide

## Abstract

**Background:** Epigenome-wide association scans (EWAS) are under way for many complex human traits, but EWAS power has not been fully assessed. We investigate power of EWAS to detect differential methylation using case-control and disease-discordant monozygotic (MZ) twin designs with genome-wide DNA methylation arrays.

**Methods and Results:** We performed simulations to estimate power under the case-control and discordant MZ twin EWAS study designs, under a range of epigenetic risk effect sizes and conditions. For example, to detect a 10% mean methylation difference between affected and unaffected subjects at a genome-wide significance threshold of *P* = 1 × 10^−6^, 98 MZ twin pairs were required to reach 80% EWAS power, and 112 cases and 112 controls pairs were needed in the case-control design. We also estimated the minimum sample size required to reach 80% EWAS power under both study designs. Our analyses highlighted several factors that significantly influenced EWAS power, including sample size, epigenetic risk effect size, the variance of DNA methylation at the locus of interest and the correlation in DNA methylation patterns within the twin sample.

**Conclusions:** We provide power estimates for array-based DNA methylation EWAS under case-control and disease-discordant MZ twin designs, and explore multiple factors that impact on EWAS power. Our results can help guide EWAS experimental design and interpretation for future epigenetic studies.

**Key Messages**

- We provide estimates of EWAS power using simulations based on DNA methylation array data.
- EWAS power was calculated under both the case-control and discordant MZ twin designs.
- We explore major factors that influence EWAS power including sample size, effect size (methylation difference and methylation odds ratio), the methylation variance of the case and control samples, and the correlation in DNA methylation between identical twin pairs.
- The provided power estimates can help guide EWAS study design and interpretation.

## Introduction

Recent advances in epigenetic technologies have enabled high-throughput epigenome-wide association scans (EWAS). To date EWAS have predominantly focused on DNA methylation, identifying many differentially methylated positions (DMPs), differentially methylated regions (DMRs) and allele-specific methylation (ASM) regions,^1^^,^^2^ related to ageing,^3–6^ environmental exposures^7–11^ and complex diseases.^12–14^ The majority of EWAS use microarray-based DNA methylation platforms, such as the Illumina Infinium HumanMethylation450 (Illumina 450K^15^) array. Several methods have recently been developed to explore epigenome-wide variation,^16–23^ but limited research has investigated power.

Similar to genome-wide association scans (GWAS), in EWAS power depends on several key factors including study design and sample size, effect size and correction for multiple testing. At least two additional factors that are specific to epigenetic data can also influence power, and these are the longitudinal stability of the epigenetic marks and their variance within a biological sample, because epigenetic signals in a biological sample from one individual represent frequency measures from a population of cells. Although most of these factors remain unknown, results from recent EWAS can provide some insights. The two most widely applied EWAS study designs to date are the case-control and the disease-discordant monozygotic (MZ) twin design, which is often sought after because twins are closely matched for genetic variation, age, sex and cohort effects, and have similar early environments. Recent EWAS findings based on these designs report modest to moderate effect sizes of 0.13% to 6.6% difference in DNA methylation levels between affected and unaffected individuals in type 1 diabetes,^24^ 10% in pain,^10^ >10% difference in systemic lupus erythematosus (SLE)^25^ and up to > 20% for environmental exposures such as smoking.^11^ To correct for multiple testing, recent EWAS have applied Bonferroni correction on the total number of regions and false-discovery rate (FDR) approaches. Longitudinal stability of epigenetic variants has been explored genome-wide, and appears to vary across regions and among individuals of different ages.^4^^,^^26^ Lastly, the impact of biological variability in epigenetic marks within a sample has been recently addressed in the context of whole blood cell composition, where it is now widely acknowledged that blood cell heterogeneity can impact on EWAS results, and computational methods have been developed to minimize these effects.^22^^,^^23^

Although power has a crucial role in EWAS, only two recent studies have addressed it in detail in the context of the case-control study design.^27^^,^^28^ In both studies, the authors estimated power under a number of assumptions and for a range samples sizes, and concluded that the distribution and variability of DNA methylation at the locus of interest can impact on power to detect small effect sizes. Greater power was attained at loci where the DNA methylation signal was less variable in both case and control groups. Furthermore, the studies also propose new measures of effect size (for example, the methylation odds ratio^27^) and extended EWAS test statistics.^28^ In the disease-discordant MZ twin design, formal power calculations are still lacking, but several studies have estimated locus-specific power estimates.^3^^,^^29–30^ These are based on different technologies and under a number of assumptions, and report a wide range of power. For example, 25 twin pairs were sufficient to reach 80% power to detect a 1.2-fold change in DNA methylation at Bonferroni correction in CpG island microarray (not single-CpG resolution) methylome data,^29^ whereas more recent examples report low (35%) to good (>80%) power to detect DMRs at single CpG sites with methylation differences of 5–6% between affected and unaffected twins in 20–22 disease-discordant twin pairs.^3^^,^^30^

Here, we estimate power of EWAS to detect the differential methylation using methylation platforms such as the Illumina 450K, under the case-control and disease-discordant twin study designs. We also evaluate the sample size required to reach 80% power under a variety of effect sizes for the two study designs. We explore factors that impact on EWAS power, such as the effect size, between-group methylation variance and the methylation correlation in twins.

## Methods

### An epigenetic model of complex disease susceptibility

**Figure 1.**
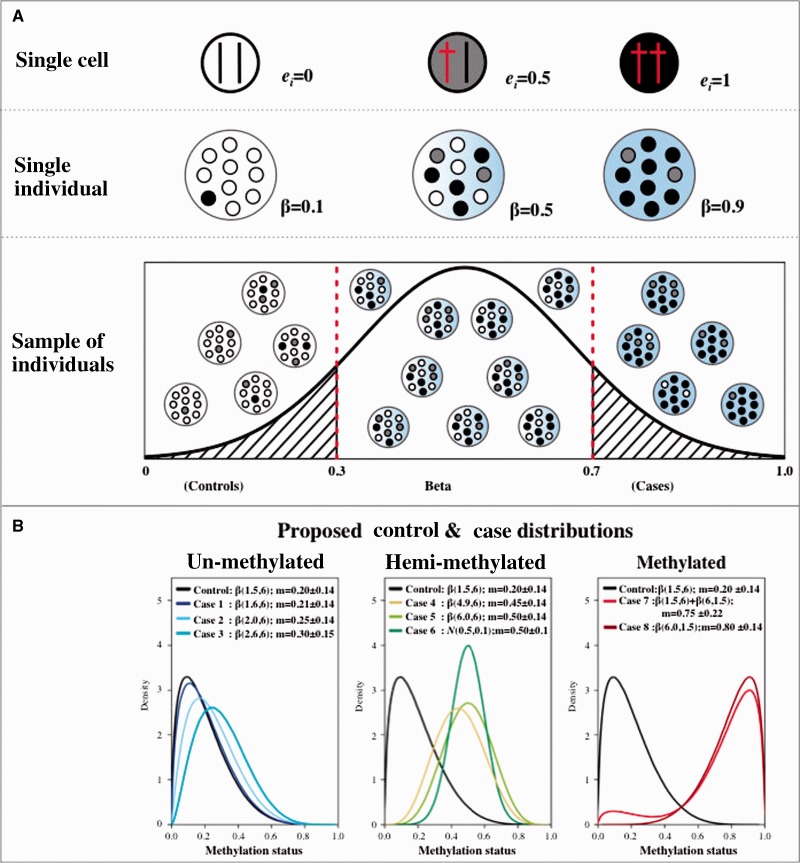
DNA methylation patterns at the (A) cellular and individual levels, and (B) with respect to the proposed methylation distributions in the simulations. We assume that a cell can have two methylated alleles (*e_i_* = 1), one methylated allele (*e_i_* = 0.5) or two unmethylated alleles (*e_i_* = 0), and one sample from an individual contains different frequencies of these cells (A, upper panel). The methylated allele is shown as a dagger symbol, and the colour of each cell represents its methylation status: un-methylated (white), hemi-methylated (grey) and methylated (black) (A, upper panel). The methylation in each sample is represented as the summary of the methylated epi-allele, denoted here as beta (A, middle panel) which can range from 0 to 1 (A, lower panel). We assume that cases have greater mean methylation levels compared with controls, and we propose one control and eight case distributions. (B) Each line represents the density of methylation levels on each proposed distribution, where the Control distribution is un-methylated, Cases 1–3 represent predominantly un-methylated samples (left panel), Cases 4–6 are hemi-methylated (middle panel) and Cases 7–8 are predominantly methylated (right panel).

We assume that disease risk is affected by DNA methylation at a single locus, *l* (Figure 1A, upper panel), where *l* represents a single CpG site in the genome. The methylation status at locus *l* in a single cell can be represented as a biallelic marker, where epi-allele 1 represents the presence of the methylated mark, and epi-allele 0 represents the absence of methylation. We assume that the disease-associated methylation mark occurs prior to onset of disease and is faithfully transmitted through mitotic cell division. We denote DNA methylation status (epi-genotype) at locus *l* as *e_j_*_,_ where the *e_j_* takes the value of 0, 0.5, and 1 to correspond to un-methylated, hemi-methylated and methylated states for a single cell. Each individual cell can consist of un-methylated, hemi-methylated and methylated epi-genotypes with probabilities of *p*1, *p*2 and *p*3, where *p*1 + *p*2 + *p*3 = 1. A sample from an individual *i* represents a population of cells (Figure 1A, middle panel), and we assume that the contribution of each cell to the population is constant and without bias. The sample-level DNA methylation estimate is a function of the methylation levels of the composition of cells (Figure 1A, lower panel), and can be described by different functions or epigenetic models. In this study, we propose a threshold model where the sample-level DNA methylation estimate reflects the allele frequency of the methylated epi-allele 1 in the cell population. That is, DNA methylation level for each sample is denoted as β (beta), which represents the sum of its fully methylated cells plus half of its hemi-methylated cells, divided by the total number of cells in the sample. In addition to the proposed DNA methylation threshold model, dominant and recessive models may also be applied, as proposed for genetic disease susceptibility risk.

### DNA methylation distribution

Multiple methods can be used to profile DNA methylation patterns across the genome. We focus on micro-array based datasets, such as those generated by the Illumina 450K array, which is currently the most widely used genome-wide technology to detect methylation in large-scale EWAS. The array measures methylated and un-methylated signals at 485 578 single CpG sites genome-wide. At each CpG site, the Illumina 450K DNA methylation level is characterized as a finite bounded quantitative trait β, calculated as:

$$Beta\;\left(\beta \right)=\frac{Methylated\;signal}{Methylated\;signal+Unmethylated\;signal+100}$$

Previous work has proposed that a single or bimodal beta distribution can be used to describe the single-locus distribution of DNA methylation levels on the Illumina 450K array.^27^ We therefore propose nine single-locus DNA methylation distributions in the context of the epigenetic disease susceptibility model. We assume that the absence of methylation is linked to the absence of disease, and propose an un-methylated distribution in unaffected individuals (Control distribution, Figure 1B), which is described by β(1.5,6) with a mean methylation level of 0.2. In our model, affected individuals will show higher levels of DNA methylation relative to controls, and we propose eight possible single-locus methylation distributions in affected individuals (Case 1–Case 8 distributions, Figure 1B). The eight case distributions have increasing ordinal mean methylation difference with the control distribution that ranges from 1% to 60% in mean DNA methylation. The eight case distributions include three distributions (Case 1–Case 3) with mean methylation levels ≤ 0.3 (un-methylated), three distributions (Case 4–Case 6) with mean methylation levels ≥ 0.45 and ≤ 0.5 (hemi-methylated) and two distributions with mean methylation levels ≥ 0.75 (methylated). The three proposed un-methylated case distributions, Case 1 to 3, follow β(1.6,6), β(2,6) and β(2.6,6) with a mean methylation level of 0.21, 0.25 and 0.30, respectively, and mean methylation difference of 1%, 5% and 10% with the control distribution, respectively. Case 4 and Case 5 characterize hemi-methylated distributions of β(4.9,6) and β(6,6) with mean methylation levels of 0.45 and 0.5, respectively, and mean methylation differences of 25% and 30%, respectively. Case 6 is also hemi-methylated, but follows the normal distribution *N*(0.5,0.1), and has the same mean methylation level as Case 5 but a smaller standard deviation. Case 7 follows the combination of 9% of β(1.5,6) and 91% of β(6,1.5) with a mean methylation of 0.75, and methylated Case 8 follows the β(6,1.5) with a mean of 0.8 that is diametrically opposed to the control distribution. The mean methylation difference between Case 7 and Case 8 with the control distribution was 55% and 60%, respectively.

### Study designs

**Figure 2.**
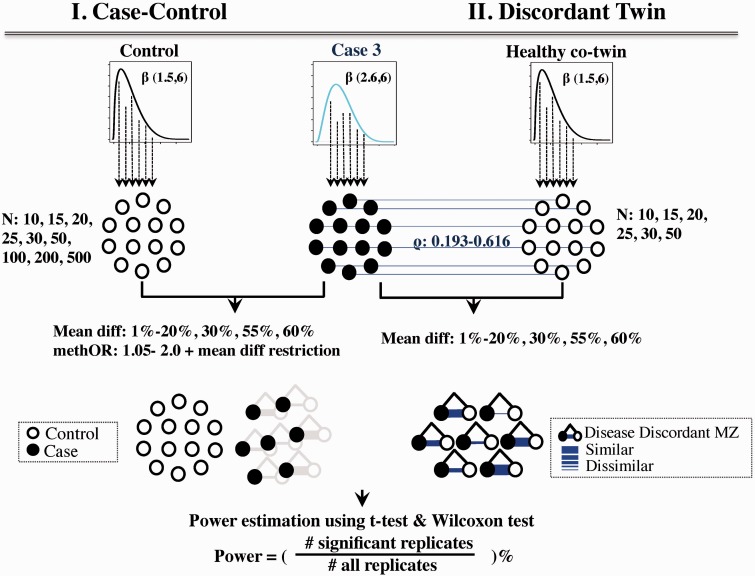
Example of a permutation procedure. Cases were drawn from the case distribution and matched controls, and healthy co-twins were drawn from the control distribution. Only permutations with a set effect size between the two groups were used in the power calculation. The cases are identical for both case-control and twin designs (black dots). Controls in the case-control design were randomly selected from the control distribution. In the twin design, DNA methylation profiles in healthy co-twin controls were correlated with cases (Spearman’s correlation coefficients between 0.193 and 0.616). The thickness of the blue line in the twin design illustrates the similarity in DNA methylation between twins.

Power was estimated under two EWAS study designs, case-control and disease-discordant monozygotic (MZ) twins. To compare power under the same parameters in the case-control and twin designs, we assumed that cases were identical in both study designs, and their matched controls and unaffected co-twins were sampled from the control distribution. In case-control design, the controls were selected based on the defined effect sizes. In the MZ discordant twin design, unaffected co-twins were selected with additional intra-pair locus-specific correlation. In each simulation, cases were selected from one of the eight Case distributions, and for the disease-discordant MZ twin design unaffected co-twins were sampled from the control distribution if: (i) the mean difference within the co-twins matched the pre-specified effect size; and (ii) the Spearman’s correlation coefficient within MZ pairs was between 0.193 and 0.616, which represented the genome-wide mean correlation coefficients ± 1 SD in a previously published set of 21 MZ twins using Illumina 27K.^3^ Once MZ twin pairs were selected, for each affected twin (or case) we also sampled a matched healthy unrelated control sample from the control distribution. Figure 2 shows an example simulation procedure by selecting the cases from distribution Case 3 and both matched unrelated controls and matched healthy co-twins from the control distribution.

### Simulation parameters

We considered case-control and disease-discordant twin samples over a range of sample sizes. As MZ twins are more difficult to recruit than unrelated cases and controls, we used a smaller sample size for the twin design, specifically 10, 15, 20, 25, 30 and 50 MZ twin pairs. Power calculations were also performed for case-control sample sizes of 10, 15, 20, 25, 30, 50, 100, 200 and 500 pairs of unrelated cases and controls (that is, altogether 20 to 1000 individuals in the sample).

As an estimate of effect size we used two approaches. First, we used the mean difference in methylation levels between affected and unaffected individuals, which ranged from 1% to 20%, 25%, 30%, 55% and 60%, and this was applied to both the twin and case-control designs. The selection of effect sizes and sample sizes was based on recently published EWAS findings as described in the introduction, and further extended to cover a broad range. In our simulation results (Supplementary Table 1a–c, available as Supplementary data at *IJE* online), we did not have power to detect effects at 1% methylation difference at single locus significance (*P* < 0.05) with 500 cases and controls, and therefore the simulations with methylation differences less than 1% were not performed. For the case-control design we also calculated effect sizes using the methylation odds ratio (methOR). Given the pre-specified range of mean methylation differences (1% to 60%), we calculated the methOR, which was previously^27^ defined as:

$$\mathrm{meth}\mathrm{OR}=\frac{\begin{array}{l} \mathrm{Mean}\;\mathrm{Methylatio}{\text{n}}_{\mathrm{Case}}\times \;(1-\mathrm{Mean}\;\mathrm{Methylatio}{\text{n}}_{\mathrm{Control}}) \end{array}}{\begin{array}{l} \left(1-\mathrm{Mean}\;\mathrm{Methylatio}{\text{n}}_{\mathrm{Case}}\right)\times \;\mathrm{Mean}\;\mathrm{Methylatio}{\text{n}}_{\mathrm{Control}} \end{array}}$$

The methOR in this study ranged from 1.05 to 2.0, and was combined with a maximum mean difference value to minimize methylation effect variability, because the range of mean differences tends to be narrower for larger samples. For example, for a methOR = 1.2, the range of mean differences is 2.63% to 3.68% in 50 case-controls, whereas the range is 2.78% to 3.38% in 500 case-controls. In this example, to reduce bias caused by variation of mean difference, we set a cutoff of 3% mean difference along with methOR = 1.2.

We estimate the variance in DNA methylation signal using the pooled standard deviation (pooled SD) of each case-control or twin sample by calculating:

$$\mathrm{Pooled}\;\mathrm{SD}\;(\text{S}{\text{D}}_{\mathrm{Case},\mathrm{Control}})=\sqrt{\frac{\begin{array}{l} \left(N_{\mathrm{Case}}-1\right)*\text{S}{\text{D}}_{\mathrm{Case}}{}^{2}+(N_{\mathrm{Control}}-1)*\text{S}{\text{D}}_{\mathrm{Control}}{}^{2} \end{array}}{\left(N_{\mathrm{Case}}+N_{\mathrm{Control}}-2\right)}}$$

We also assessed the correlation in DNA methylation profiles between cases and controls, and between affected twins and healthy co-twins. We calculated between-group correlation using Spearman’s correlation coefficients (ρ).

The statistical significance threshold was set at a *P*-value threshold of 0.05 for single locus analysis, and a *P*-value threshold of 1 × 10^−6^ for genome-wide significance. This threshold was selected using Bonferroni correction based on a subset of the probes on the Illumina 450K array, because some regions show evidence for co-methylation. Furthermore, recent EWAS using Illumina 450K data have reported FDR-based thresholds of 1% to 5% FDR with corresponding *P*-values close to *P* = 1 × 10^−4^.^31^^,^^32^

### Estimation of statistical power

Power estimation was based on simulations. For the parametric analyses, a t test with a prior F test for equal variance was performed in the case-control design and a paired t test was performed in the twin study design. All of the case-control simulations include equal and unequal variances between cases and controls with the exception of one case-multiple control scenario with a greater proportion of unequal variances. Supplementary Tables 1a–c show results from simulations with equal variances between cases and controls. The corresponding nonparametric analyses, Wilcoxon rank sum test (also called the Mann–Whitney U test) and Wilcoxon signed rank test, were also performed. All statistical analyses were performed in R version 2.15.0.

## Results

### Power of case-control EWAS using mean difference effect estimates

**Figure 3.**
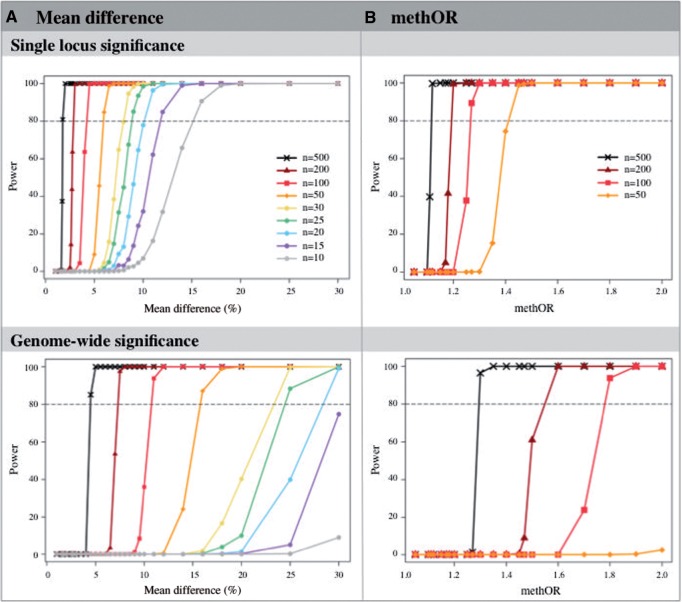
Power of case-control EWAS. Estimates are obtained for a range of sample sizes, using (A) mean difference and (B) methOR effects, at nominal (upper panel) and genome-wide (lower panel) significance thresholds. Each line represents the power curve under different case-control sample sizes from 10 to 500 pairs of cases and controls.

Power simulations were performed under the case-control EWAS design, by sampling effect sizes based on the mean difference in DNA methylation between cases and controls. Cases were selected from one of eight case distributions and controls were drawn from the control distribution, using 1000 permutations per simulation. Simulations were performed with mean difference effects from 1% to 60% and with increasing sample sizes from 10 to 500 pairs of cases and controls, that is, 20 to 1000 individuals altogether (Figure 3A, Supplementary Table 1a, available as Supplementary data at *IJE* online). Figure 3A shows the mean difference required to achieve 80% power at different sample sizes at *P*-value thresholds of 0.05 (single locus threshold, upper plot) and 1 × 10^−6^ (genome-wide threshold, lower plot). For example, a sample size of 100 cases and 100 controls results in over 80% power to detect a 4.5% mean difference (mean methOR = 1.32) in methylation at nominal significance (*P* = 0.05). However, at a genome-wide significance (*P* = 1 × 10^−6^) the same sample size gives over 80% power to detect a much larger effect size of 11% mean difference (mean methOR = 1.81). The results of the Wilcoxon test are shown in Supplementary Table 1 and 2, available as Supplementary data at *IJE* online. We also performed power estimation under the one case-multiple controls scenario. We show results from one case:two controls and one case:four controls study design (Supplementary Table 1b and c, available as Supplementary data at *IJE* online) and, as expected, power increases when the sample size of the control group increases.

### Power of case-control EWAS using methOR effect estimates

We next considered using the methOR as a measure of effect size in the case-control design. Power estimates were obtained from simulations with methOR effects of 1.05 to 2.0 and with increasing sample sizes from 50 to 500 pairs of cases and controls (Figure 3B, Supplementary Table 2 available as Supplementary data at *IJE* online). To achieve 80% power to detect differential methylation at nominal significance, the minimum methOR that could be detected ranged from 1.15 for a sample of 500 cases and 500 controls, to 1.45 for a sample of 50 cases and 50 controls. To achieve 80% power to detect differential methylation at genome-wide significance, sample sizes of at least 100 cases and 100 controls were required to detect methORs of at least 1.8, and no power was observed for smaller samples (*n* ≤ 50 cases and 50 controls).

### Power of discordant twin EWAS

**Figure 4.**
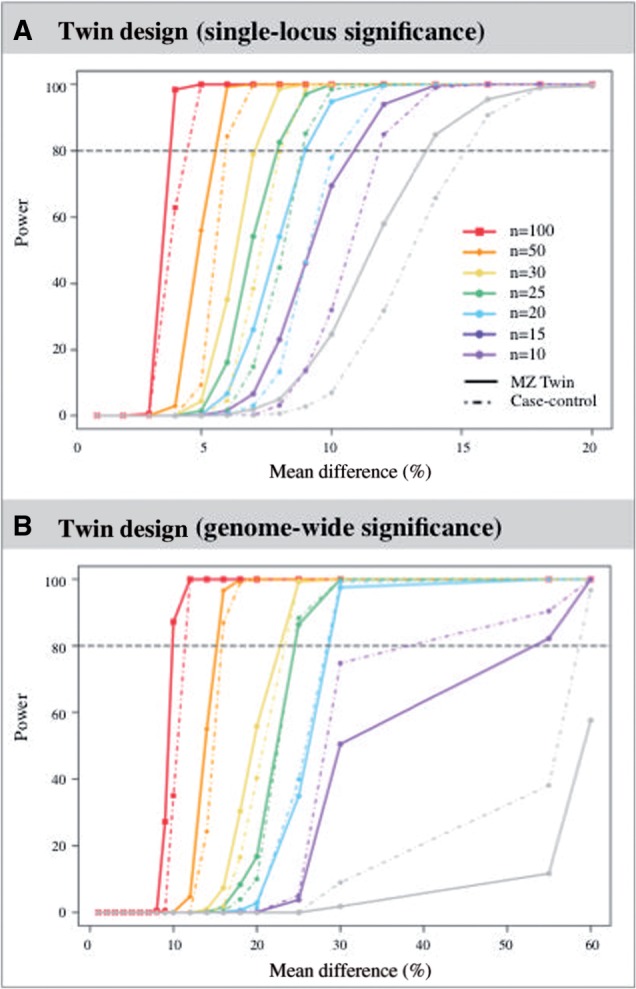
Power of discordant twin EWAS. Estimates are shown for the twin (solid lines) and case-control (dashed lines) designs for a range of sample sizes and mean differences at a significance level of 0.05 (A, upper panel) and 1 × 10^−6^ (B, lower panel). Each line represents the power curve under different sample sizes from 10 to 100 pairs of twins, or pairs of cases and controls.

We next estimated EWAS power under the disease-discordant MZ twin design. Simulations were performed with mean difference effects from 1% to 60% and with sample sizes of 10, 15, 20, 25, 30 and 50 twin pairs (Table 1, Figure 4). For example, we observed that a sample of 25 twin pairs has over 80% power to detect a mean difference of 8% in methylation at nominal significance (*P* = 0.05), and 25% at genome-wide significance (*P* = 1 × 10^−6^). As expected, power estimates in twins outperformed the case-control design (Table 1, Figure 4). For example, a sample of 25 twin pairs has over 80% power to detect a mean difference of 8% in methylation at nominal significance (*P* = 0.05), whereas 25 pairs of cases and controls have only 45% power to detect this effect (Figure 4A). At genome-wide significance, at least 50 pairs of subjects were required to identify effect sizes of 16% mean difference with over 80% power in both designs (Figure 4B). However, our simulations were not designed for a formal comparison between case-control and twin power, because our results assume that twins and case-control samples are equally well matched for factors that can influence differential methylation, including age, sex and cohort effects, and unrelated samples are typically more heterogeneous than MZ twins.

**Table 1.** Power of EWAS twin and case-control designs

Diff	*n* = 10					*n* = 15					*n* = 20				*n* = 25				*n* = 30				*n* = 50			
	TWIN			CACO		TWIN		CACO			TWIN		CACO		TWIN		CACO		TWIN		CACO		TWIN		CACO	
	*P* < 0.05	*P* < 10^−6^	*P* < 0.05		*P* < 10^−6^	*P < 0.05*	*P < 10^−6^*		*P < 0.05*	*P < 10^−6^*	*P* < 0.05	*P* < 10^−6^	*P* < 0.05	*P* < 10^−6^	*P* < 0.05	*P* < 10^−6^	*P* < 0.05	*P* < 10^−6^	*P* < 0.05	*P* < 10^−6^	*P* < 0.05	*P* < 10^−6^	*P* < 0.05	*P* < 10^−6^	*P* < 0.05	*P* < 10^−6^
1%	0	0	0		0	0	0		0	0	0	0	0	0	0	0	0	0	0	0	0	0	0	0	0	0
2%	0	0	0		0	0	0		0	0	0	0	0	0	0	0	0	0	0	0	0	0	0	0	0	0
3%	0	0	0		0	0	0		0	0	0	0	0	0	0	0	0	0	0	0	0	0	0	0	0	0
4%	0	0	0		0	0	0		0	0	0	0	0	0	0	0	0	0	0	0	0	0	2.9	0	0	0
5%	0	0	0		0	0.1	0		0	0	0.2	0	0.1	0	1.4	0	0	0	4.4	0	0	0	56.0	0	9.7	0
6%	0.3	0	0		0	1.5	0		0.1	0	6.7	0	0.6	0	16.1	0	1.1	0	35.2	0	3.5	0	99.3	0	85.3	0
7%	1.9	0	0.3		0	6.7	0		0.6	0	26.1	0	3.7	0	54.2	0	15.4	0	79.0	0	37.7	0	100	0	99.9	0
8%	5.0	0	0.8		0	23.1	0		3.9	0	54.1	0	17	0	82.5	0	45.3	0	98.6	0	80.8	0	100	0	100	0
9%	13.5	0	2.2		0	46.2	0		12.7	0	80.4	0	47.1	0	97	0	85.1	0	100	0	99.4	0	100	0.1	100	0
10%	24.7	0	8.4		0	69.5	0		36	0	94.8	0	80.1	0	100	0	98.4	0	100	0	100	0	100	0.1	100	0
12%	58.0	0	31.4		0	94.0	0		83.6	0	99.8	0	99.5	0	100	0	100	0	100	0	100	0	100	4.8	100	0.6
14%	84.8	0	66.8		0	99.7	0		99	0	100	0	100	0	100	0.1	100	0	100	0.9	100	0.1	100	55.0	100	25.6
16%	95.5	0	83.3		0	100	0		100	0	100	0.2	100	0	100	1.7	100	0	100	7.4	100	0.3	100	99.9	100	70
18%	99.0	0	97.6		0	100	0		100	0	100	0.7	100	0.1	100	8.4	100	0.3	100	30.4	100	6.8	100	100	100	99.5
20%	99.5	0	99.9		0	100	0.1		100	0.1	100	2.8	100	1.2	100	16.8	100	10.3	100	55.8	100	42.5	100	100	100	100
25%	100	0	100		0.1	100	3.8		100	4	100	34.9	100	38.8	100	86.5	100	90.5	100	99.3	100	99.4	100	100	100	100
30%	100	0.5	100		3	100	25.2		100	38.6	100	86.8	100	94.6	100	100	100	100	100	100	100	100	100	100	100	100
30%	100	1.8	100		13.2	100	50.5		100	81.7	100	97.6	100	100	100	100	100	100	100	100	100	100	100	100	100	100
55%	100	11.7	100		52.7	100	82.2		100	99.6	100	100	100	100	100	100	100	100	100	100	100	100	100	100	100	100
60%	100	57.6	100		98.4	100	100		100	100	100	100	100	100	100	100	100	100	100	100	100	100	100	100	100	100

Diff, mean methylation difference between affected and unaffected individuals; *n*, sample size (twin pairs or pairs of cases and controls), TWIN, discordant MZ twin design (paired t test); CACO, case-control design (two-sample t test).

### Sample size required to reach 80% power in EWAS twin and case-control designs

We estimated the sample size required to reach 80% power in both the twin and case-control designs (Table 2). Effects were simulated using mean differences of 7% to 15% for both case-controls and twins. Power was estimated at nominal significance (*P* = 0.05) and at a EWAS genome-wide significance threshold of *P* = 1 × 10^−6^. Twins required a smaller sample size to reach 80% power compared with case-controls. In general, the sample sizes required to detect larger mean differences (≥13%) were similar between twins and case-controls, but differed when mean differences were smaller (≤10%). For example, to detect a mean difference of 7% at genome-wide significance, 178 pairs of twins were required and 211 cases and 211 controls were needed. Similar sample sizes were estimated using the nonparametric Wilcoxon test.

**Table 2.** Sample size requirements for 80% power in EWAS twin and case-control designs

Diff	Twin				Case-control			
	*P* < 0.05		*P* < 1 × 10^−6^		*P* < 0.05		*P* < 1 × 10^−6^	
	t-test^a^	Wilcox^b^	t-test^a^	Wilcox^b^	t-test^c^	Wilcox^d^	t-test^c^	Wilcox^d^
7%	30	30	178	178	37	37	211	211
8%	25	25	145	149	30	30	169	169
9%	20	20	117	117	24	24	137	137
10%	17	18	98	102	20	21	112	110
11%	15	15	81	83	17	18	96	95
12%	13	13	71	71	15	16	80	80
13%	11	12	63	69	13	13	70	70
14%	10	11	55	62	11	13	61	63
15%	9	10	50	57	10	11	54	57

Diff, mean methylation difference between affected and unaffected individuals.

^a^t test, paired t test.

^b^Wilcox, Wilcoxon signed-rank test.

^c^t test, two-sample t test.

^d^Wilcox, Wilcoxon rank-sum test.

### DNA methylation variance can impact on power in the EWAS case-control design

**Figure 5.**
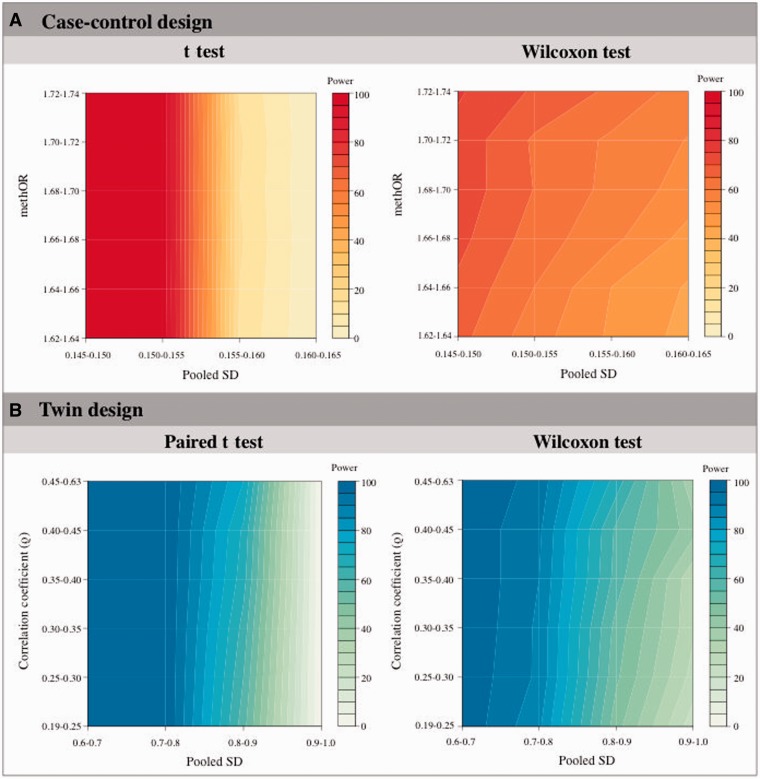
DNA methylation variance and correlation can impact EWAS power. Case-control power estimates (A, upper panel) are shown under different pooled SDs and methORs at a fixed mean difference = 10% using parametric (left panel) and nonparametric (right panel) test statistics. MZ twin power estimates (B, lower panel) are shown under different pooled SDs and correlation coefficients at a fixed mean difference = 9% using parametric (left panel) and nonparametric (right panel) test statistics.

We explored the effect of DNA methylation variance on EWAS power by estimating the pooled SD in DNA methylation for the combined case-control sample as a measure of variance (Figure 5A). We selected permutations with 20 cases and 20 controls at a 10% methylation mean difference and with equal variances, and estimated power by categorizing the pooled SD into four groups (0.145–0.150, 0.150–0.155, 0.155–0.160 and 0.160–0.165) and methOR into six groups (1.62–1.64, 1.64–1.66, 1.66–1.68, 1.68–1.70, 1.70–1.72 and 1.72–1.74). Power was estimated using the t test (Figure 5A, left panel) and Wilcoxon test (Figure 5A, right panel) at nominal significance. Under the t test, the pooled SD greatly influences power where greater pooled SD will lead to lower power irrespective of methOR differences. In comparison, both pooled SD and methOR have an influence on power estimated using the Wilcoxon test. Greatest power can be achieved with smaller pooled SD and at highest methOR.

To further explore the influence of methylation variance on power, we selected permutations with the same 20 cases and 20 controls at a 10% methylation mean difference, but only using simulations where the variance of cases was not equal to that of the controls. The major difference between the equal and unequal variance t test is in the denominator of the t statistic and degrees of freedom. In the unequal variance test, the variance between groups was calculated by:

$$\text{S}{\text{D}}_{\overset{�}{\mathrm{Case}}-\overset{�}{\mathrm{Control}}}=\sqrt{\frac{\text{S}{\text{D}}_{\mathrm{Case}}{}^{2}}{N_{\mathrm{Case}}}+\frac{\text{S}D_{\mathrm{Control}}{}^{2}}{N_{\mathrm{Control}}}}$$

Power estimates in the unequal variance case-control simulations were categorized using this pooled standard deviation into four groups (0.040–0.042, 0.042–0.044, 0.044–0.046 and 0.046–0.048), and using methOR into six groups (1.62–1.64, 1.64–1.66, 1.66–1.68, 1.68–1.70, 1.70–1.72 and 1.72–1.74). Furthermore, we also considered which group (cases or controls) had the greater variance; that is, either the variance in cases was greater than that in controls, or the variance in cases was smaller than that in controls. Compared with the simulations with equal variances between the groups, the power estimations from the unequal variance results were quite similar for the t test (Supplementary Figure 1, left panel, available as Supplementary data at *IJE* online). It is easier to reach greater power when the variance in the cases is smaller than that in controls, and a more distinct pattern is found using the Wilcoxon test under the same parameter settings (Supplementary Figure 1, right panel, available as Supplementary data at *IJE* online). Similarly to the equal variance results, the methOR and pooled variance impact on power (Supplementary Figure 2, available as Supplementary data at *IJE* online). These results also highlight the importance of choosing the appropriate analytical method across the equal variance t test, the unequal variance t test and the Wilcoxon test.

### DNA methylation variance and twin correlation can influence power in the EWAS twin design

The impact of methylation variance on power in the case-control design suggests that similar effects may exist in the twin design. We therefore assessed power in the EWAS twin design by considering the pooled SD of the DNA methylation signal in the twin sample, as well as the correlation in methylation between co-twins (Figure 5B). We performed permutations by varying the pooled SD and correlation, at a set methylation difference of 9% in 20 pairs of twins. Because 9% methylation difference can correspond to a range of methORs (from 1.30 to 2.44) in the case-control design, which can also impact on power, we further restricted the permutations to give a set methOR = 1.67. Power was estimated at nominal significance by categorizing pooled SD into four groups (0.6–0.7, 0.7–0.8, 0.8–0.9 and 0.9–1.0), and the correlation into six groups (0.19–0.25, 0.25–0.30, 0.30–0.35, 0.35–0.40, 0.40–0.45 and 0.45–0.63). The smallest pooled SD results in greatest power and, under the same pooled SD, permutations with higher twin correlation result in greater power. Compared with the t test, the Wilcoxon test gives slightly lower power under moderate pooled SD, but the Wilcoxon test can outperform the t test under larger pooled SD. Smaller pooled SD, greater mean difference and greater Spearman’s correlation within twins can result in greater power.

## Discussion

Statistical power and sample size are crucial to EWAS design and interpretation. Here, we estimate power and sample size limitations for two most commonly applied EWAS designs under a number of key assumptions. EWAS power has previously been explored in the case-control context, but our results provide a first characterization of power for the disease-discordant MZ twins EWAS design across a range of epigenetic disease models.

MZ twins share nearly all of their genetic variants, are matched for age, gender and cohort effects, and have similar *in utero* and maternal effects and many early-life environmental factors. All of these factors have either been shown or are hypothesized to influence DNA methylation levels throughout the genome. Therefore, MZ twins are a much more homogeneous sample relative to genetically heterogeneous unrelated individuals who are exposed to different environments throughout life, and correspondingly MZ twins have been shown to have much more similar levels of DNA methylation compared with dizygotic (DZ) co-twins and unrelated pairs of individuals.^3^^,^^33^ It is difficult to incorporate all of these factors in a simulation study, therefore in an attempt to minimize some of these effects, we assumed that all individuals in our study were the same age and gender and were exposed to similar cohort effects. This will bias the case-control sample towards homogeneity and may give inflated power estimates for the case-control design. Therefore, we cannot directly compare the power estimates of case-control and twin designs. The EWAS case-control and EWAS twin designs are complementary to each other and can be used jointly to identify the cause of the identified disease-associated DMR. The twin design can be used to identify disease-related DMRs that are either caused by stochastic or environmental factors, or that are a consequence of the disease. In contrast, samples of unrelated individuals provide the option to integrate genetic and DNA methylation datasets to explore potential genetic impacts on the trait that are mediated by methylation.

Our findings build on two previous studies that explore power in the case-control design.^27^^,^^28^ In general, the case-control power estimates and conclusions are consistent with previous results.^27^^,^^28^ For example, using 200 cases and 200 controls, a methOR of 1.49 and a mean difference of 7.2% previously resulted in 16% power under the Wald test.^27^ The closest scenarios in our study were using 200 cases and 200 controls, simulating a methOR between 1.45 (mean difference = 6.4%) and up to methOR of 1.5 (mean difference = 7.0%), which resulted in power between 18% and 67% under the Wilcoxon test, respectively. Although power is close to previous estimates, there is a divergence which could be explained by the different composition of the underlying DNA distributions. Both previous power studies proposed that the single-locus DNA methylation distribution is composed by a mixture distribution, either a Uniform-Normal mixture^28^ or single or combined Beta distributions,^27^ whereas we assumed that the cases follow predominantly a single Beta distribution and the controls remained un-methylated. Our assumption was based on published profiles from 172 healthy female subjects^3^ measured by the Illumina 27K array, where 69% (*n* = 24 641) of the autosomal CpGs were un-methylated and the majority of methylation distributions on each locus followed single Beta distribution with small standard deviation (85% of probes with SD < 0.05). Therefore, as previously noted, the shape of the underlying single-locus DNA methylation distribution will play a role in power.

One of the major characteristics of the DNA methylation distribution is the variance in DNA methylation. DNA methylation variance has previously been shown to impact power,^28^ and we confirm these results not only in the case-control design but also in the twin EWAS design. Another conclusion that is consistent across studies^27^ is that the methOR measure of epigenetic effect appears to correlate better with power than the mean difference effect. Finally, the similarity in DNA methylation profiles within pairs of genetically identical twins can impact on EWAS power in the twin design.

Some of the limitations of our study arise from the major assumptions. One of these is that DNA methylation occurs prior to disease onset and is mitotically stable. Recent genome-wide data on longitudinal stability of DNA methylation marks show that there is great variability in longitudinal stability of methylation marks across the genome and with respect to age of the individual.^4^ Another key assumption is that we explore DNA methylation profiles in the tissue that is most relevant to the disease. For many diseases, access to clinically relevant tissues is not feasible and surrogates such as whole blood are often used in EWAS. Both tissue-shared and tissue-specific DNA methylation profiles exist across the genome, and modelling these effects in our epigenetic disease susceptibility models is difficult with limited empirical data. A third overly simplistic assumption is to model the similarity in DNA methylation profiles within MZ twins as a range of correlations from empirical estimates.^3^ Several reports have identified and replicated twin-based DNA methylation heritable regions in the genome,^3^^,^^31^^,^^33^ and have clearly shown that MZ twins have more similar methylation profiles than unrelated individuals.^3^ However, the precise structure of this correlation along the genome varies.^31^ Lastly, we considered power and sample size estimates under models where a single CpG site is associated with the phenotype. It is possible that multiple CpG sites impact on the phenotype, either as an epi-haplotype (where taking into account co-methylation may be informative), or under models of CpG-interaction. For many of these assumptions, the relevant parameters are difficult to estimate because of lack of in-depth data.

In summary, using comprehensive power calculations we provide power limits of EWAS for the case-control and discordant twin designs under a range of models and several key assumptions. Our findings can help EWAS design and interpretation.

## Supplementary Data

Supplementary data are available at *IJE* online.

## Funding

Support for this work was obtained from: the Wellcome Trust (082713/Z/07/Z), the European Research Council (ERC 250157) and in part from TwinsUK, which is funded by the Wellcome Trust; the European Community’s Seventh Framework Programme (FP7/2007-2013); and the National Institute for Health Research (NIHR) BioResource, Clinical Research Facility and Biomedical Research Centre based at Guy's and St Thomas' NHS Foundation Trust and King's College London.

**Conflict of interest:** None declared.

## Supplementary Material

Supplementary Data
