# Supplementary material for: Power and sample size estimation for epigenome-wide association scans to detect differential DNA methylation
Source: Int J Epidemiol. 2015 May 12;44(4):1429–41. doi: 10.1093/ije/dyv041 (PMC4588864; doi:10.1093/ije/dyv041)
Supplement: Supplementary Data [file supp_44_4_1429__index.html]

Power and sample size estimation for epigenome-wide association scans to detect differential DNA methylation — Power and sample size estimation for epigenome-wide association scans to detect differential DNA methylation — Supplementary Data 

# Power and sample size estimation for epigenome-wide association scans to detect differential DNA methylation

## Supplementary Data

files

**Files in this Data Supplement:**

- Supplementary Data - tiff file
- Supplementary Data - tiff file
- Supplementary Data - xlsx file
- Supplementary Data - xlsx file
- Supplementary Data - xlsx file
- Supplementary Data - docx file
