# Supplementary figures and images for: Power and sample size estimation for epigenome-wide association scans to detect differential DNA methylation
Source: Int J Epidemiol. 2015 May 12;44(4):1429–41. doi: 10.1093/ije/dyv041 (PMC4588864; doi:10.1093/ije/dyv041)

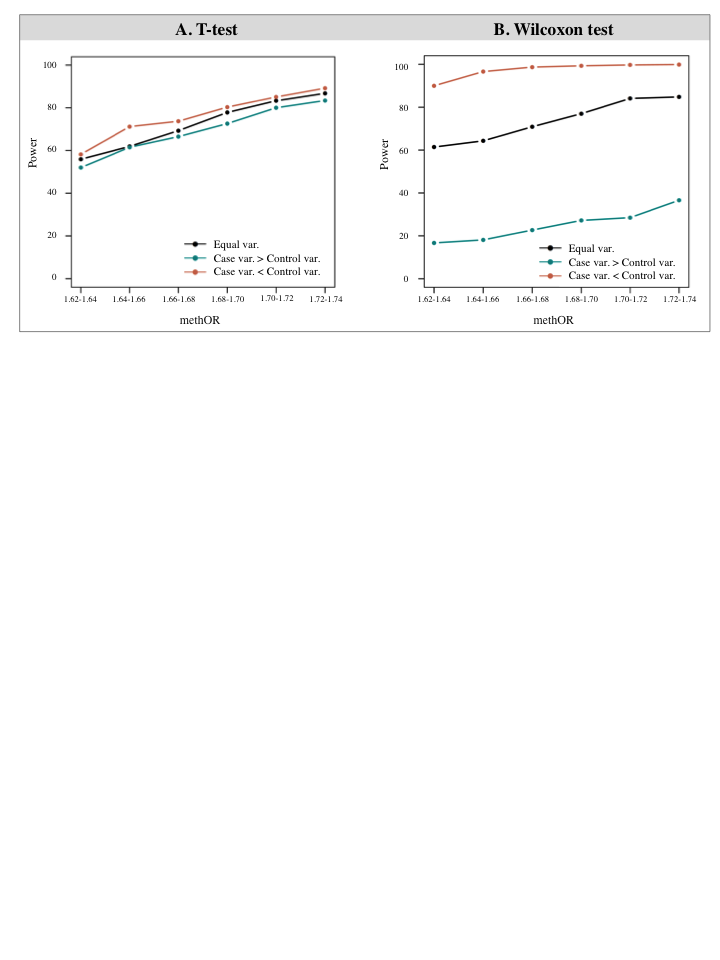

Supplement: Supplementary Data [file supp_dyv041_dyv041Supplementary_Figure_1.tif]

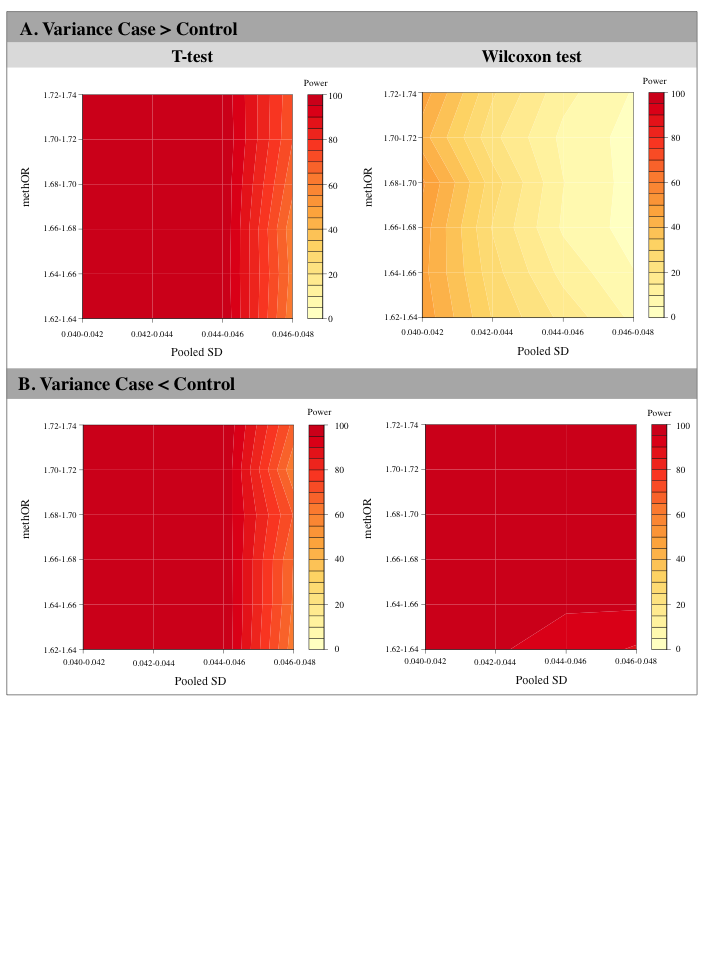

Supplement: Supplementary Data [file supp_dyv041_dyv041Supplementary_Figure_2.tif]
