# Supplementary material for: Power and sample size estimation for epigenome-wide association scans to detect differential DNA methylation
Source: Int J Epidemiol. 2015 May 12;44(4):1429–41. doi: 10.1093/ije/dyv041 (PMC4588864; doi:10.1093/ije/dyv041)
Supplement: Supplementary Data [file supp_dyv041_dyv041Supplementary_Table_2.docx]

**Supplementary Table 2. Power of large-scale case-control EWAS using methOR effects.**

| methOR/ diff ^1^ | D^2^ | ***N=50*** | | | | |  | ***N=100*** | | | | | |  | ***N=200*** | | | | | | | | | | |  | | ***N=500*** | | | | | | | | | | |
| --- | --- | --- | --- | --- | --- | --- | --- | --- | --- | --- | --- | --- | --- | --- | --- | --- | --- | --- | --- | --- | --- | --- | --- | --- | --- | --- | --- | --- | --- | --- | --- | --- | --- | --- | --- | --- | --- | --- |
|  |  | Mean diff (range)^3^ | P < 0.05 | | P < 1×10^-6^ | |  | Mean diff (range) | P < 0.05 | | | P < 1×10^-6^ | |  | Mean diff (range) | | P < 0.05 | | | | | P < 1×10^-6^ | | | |  | | Mean diff (range) | | P < 0.05 | | | P < 1×10^-6^ | | | | | |
|  |  |  | T^4^ | W^5^ | T | W |  |  | T | W | T | | W |  |  |  | T | | W | | T | | | W | |  | |  |  | T | | W | | | T | | W | |
| 1.05, <1% | C1 | 0.79 (0.65, 0.98) | 0 | 0 | 0 | 0 |  | 0.80 (0.67, 0.92) | 0 | 0 | 0 | | 0 |  | 0.80 (0.68, 0.92) | | 0 | | 0 | | 0 | | | 0 | |  | | 0.800 (0.7, 0.89) | | 0 | | 0 | | | 0 | | 0 | |
| 1.10, <2% | C1 | 1.55 (1.31, 1.87) | 0 | 0 | 0 | 0 |  | 1.55 (1.33, 1.76) | 0 | 0 | 0 | | 0 |  | 1.55 (1.39, 1.76) | | 0 | | 0 | | 0 | | | 0 | |  | | 1.55 (1.42, 1.69) | | 0 | | 33.0 | | | 0 | | 0 | |
| 1.11, <2% | C1 | 1.69 (1.38, 1.98) | 0 | 0 | 0 | 0 |  | 1.70 (1.49, 1.93) | 0 | 0 | 0 | | 0 |  | 1.70 (1.53, 1.88) | | 0 | | 1.1 | | 0 | | | 0 | |  | | 1.70 (1.58, 1.84) | | 39.7 | | 53.4 | | | 0 | | 0 | |
| 1.15, <2.5% | C1 | 2.26 (1.94, 2.50) | 0 | 0 | 0 | 0 |  | 2.27 (1.99, 2.50) | 0 | 0.4 | 0 | | 0 |  | 2.28 (2.07, 2.49) | | 0 | | 13.5 | | 0 | | | 0 | |  | | 2.27 (2.12, 2.44) | | 100 | | 98.9 | | | 0 | | 0 | |
| 1.20, <3% | C2 | 2.92 (2.63, 3.00) | 0 | 0.7 | 0 | 0 |  | 2.90 (2.64, 3.00) | 0 | 5.8 | 0 | | 0 |  | 2.92 (2.72, 3.00) | 99.4 | | 69.8 | | 0 | | | 0 | |  | | 2.94 (2.78, 3.00) | | 100 | | 100 | | | 0 | | 0 | |  |
| 1.25, <4% | C2 | 3.81 (3.37, 4.00) | 0 | 3.5 | 0 | 0 |  | 3.83 (3.45, 3.99) | 37.7 | 65.7 | 0 | | 0 |  | 3.87 (3.58, 3.99) | | 100 | | 100 | | 0 | | | 0 | |  | | 3.88 (3.67, 3.99) | | 100 | | 100 | | | 0 | | 19.9 | |
| 1.30, <5% | C2 | 4.55 (3.80, 5.00) | 0.2 | 17.2 | 0 | 0 |  | 4.56 (4.08, 4.95) | 99.9 | 94.2 | 0 | | 0 |  | 4.55 (4.26, 4.88) | | 100 | | 100 | | 0 | | | 0 | |  | | 4.56 (4.24, 4.83) | | 100 | | 100 | | | 96.5 | | 97.4 | |
| 1.35, <5.5% | C2 | 5.17 (4.51, 5.50) | 15.3 | 45.5 | 0 | 0 |  | 5.20 (4.65, 5.50) | 100 | 99.5 | 0 | | 0 |  | 5.21 (4.83, 5.50) | | 100 | | 100 | | 0 | | | 0.1 | |  | | 5.22 (4.96, 5.49) | | 100 | | 100 | | | 100 | | 100 | |
| 1.40, <6% | C2 | 5.70 (4.89, 6.00) | 74.5 | 70.8 | 0 | 0 |  | 5.77 (5.29, 5.99) | 100 | 100 | 0 | | 0 |  | 5.81 (5.25, 5.99) | | 100 | | 100 | | 0 | | | 0.8 | |  | | 5.83 (5.54, 5.99) | | 100 | | 100 | | | 100 | | 100 | |
| 1.45, <6.5% | C2 | 6.24 (5.58, 6.50) | 98.8 | 88.8 | 0 | 0 |  | 6.31 (5.70, 6.50) | 100 | 100 | 0 | | 0 |  | 6.36 (5.93, 6.50) | | 100 | | 100 | | 0.5 | | | 18.3 | |  | | 6.39 (6.04, 6.50) | | 100 | | 100 | | | 100 | | 100 | |
| 1.50, <7.5% | C2 | 6.97 (5.87, 7.50) | 100 | 97.2 | 0 | 0 |  | 7.00 (6.24, 7.49) | 100 | 100 | 0 | | 0 |  | 7.01 (6.51, 7.46) | | 100 | | 100 | | 61.0 | | | 67.4 | |  | | 7.01 (6.74, 7.36) | | 100 | | 100 | | | 100 | | 100 | |
| 1.60, <9% | C3 | 8.61 (7.35, 9.00) | 100 | 100 | 0 | 0 |  | 8.70 (2.95, 8.99) | 100 | 100 | 0 | | 1 |  | 8.73 (8.19, 8.99) | | 100 | | 100 | | 100 | | | 100 | |  | | 8.77 (8.43, 8.99) | | 100 | | 100 | | | 100 | | 100 | |
| 1.70, <10% | C3 | 9.64 (8.43, 10.00) | 100 | 100 | 0 | 0 |  | 9.73 (8.93, 9.99) | 100 | 100 | 23.9 | | 40.7 |  | 9.79 (9.08, 9.99) | | 100 | | 100 | | 100 | | | 100 | |  | | 9.84 (9.38, 9.99) | | 100 | | 100 | | | 100 | | 100 | |
| 1.80, <11% | C3 | 10.62 (9.50, 11.00) | 100 | 100 | 0 | 0 |  | 10.72 (9.90, 10.99) | 100 | 100 | 93.8 | | 91.8 |  | 10.79 (10.20, 10.99) | | 100 | | 100 | | 100 | | | 100 | |  | | 10.84 (10.40, 10.99) | | 100 | | 100 | | | 100 | | 100 | |
| 1.90, <12% | C3 | 11.59 (10.24, 12.00) | 100 | 100 | 0.3 | 0.2 |  | 11.69 (10.79, 11.99) | 100 | 100 | 100 | | 99.8 |  | 11.76 (11.12, 11.99) | | 100 | | 100 | | 100 | | | 100 | |  | | 11.83 (11.46, 11.99) | | 100 | | 100 | | | 100 | | 100 | |
| 2.00, <14% | C3 | 12.76 (11.12, 13.97) | 100 | 100 | 2.5 | 1.3 |  | 12.80 (11.71, 13.70) | 100 | 100 | 100 | | 100 |  | 12.79 (12.00, 13.63) | | 100 | | 100 | | 100 | | | 100 | |  | | 12.78 (12.22, 13.30) | | 100 | | 100 | | | 100 | | 100 | |

^1^methOR/diff: Mean methylation odds ratio and set value of mean difference between cases and controls; ^2^D: Case distributions of sample draw, C1 to C3 corresponding to case distribution 1 to case distribution 3; ^3^Mean diff: Mean methylation difference between cases and controls; ^4^T: two sample t-test; ^5^W: Wilcoxon rank-sum test
